# Influence of Vine Shoot Waste and Its Derived Ash on the Properties of Cement Composites

**DOI:** 10.3390/molecules30234560

**Published:** 2025-11-26

**Authors:** Daniela Alexandra Scurtu, Erika Andrea Levei, Eniko Kovacs, Lacrimioara Senila, Oana Cadar, Dorina Simedru, Cecilia Roman, Xenia Filip, Leontin David

**Affiliations:** 1 Research Institute for Analytical Instrumentation Subsidiary, National Institute of Research and Development for Optoelectronics INOE 2000, 67 Donath Street, 400293 Cluj-Napoca, Romania; daniela.scurtu@icia.ro (D.A.S.); lacri.senila@icia.ro (L.S.); oana.cadar@icia.ro (O.C.); dorina.simedru@icia.ro (D.S.); cecilia.roman@icia.ro (C.R.); leontin.david@ubbcluj.ro (L.D.); 2 National Institute for Research and Development of Isotopic and Molecular Technologies, 67-103 Donath Street, 400293 Cluj-Napoca, Romania; xenia.filip@itim-cj.ro; 3 Faculty of Physics, Babes-Bolyai University, 1 Mihail Kogalniceanu Street, 400084 Cluj-Napoca, Romania

**Keywords:** vine shoot waste ash, cement composite, NMR spectroscopy, mechanical properties

## Abstract

The valorization of agricultural byproducts plays a critical role in advancing the circular economy. Vine cultivation produces significant amounts of biomass waste throughout the year, posing environmental challenges if left unmanaged. This study investigates the potential reuse of vine shoot waste and its derived ash as alternative components in cement-based materials. The properties of the composites containing 1% vine shoot waste or its derived ash incorporated in cement paste were comparatively assessed with those of cement paste prepared from Portland cement based on the Fourier-transform infrared spectra, X-ray diffraction patterns, scanning electron microscopy images, EDX elemental maps, and solid-state ^27^Al and ^29^Si nuclear magnetic resonance spectra and thermal analysis. Mechanical performance was assessed through flexural and compressive strength tests. The results confirm the potential of vine shoot waste use as a sustainable additive in cementitious materials and its contribution to reducing the environmental footprint of the cement industry.

## 1. Introduction

Cement is an essential material in the construction industry, but its production is energy-intensive and generates significant CO_2_ emissions, posing serious environmental challenges. Consequently, there is growing interest in developing sustainable alternatives and adopting greener practices [1]. A promising strategy involves the development of new cement composites by the incorporation of natural and waste-derived materials. Mineral additives such as fly ash, industrial by-products, and various waste types can partially replace cement and contribute to a more sustainable construction sector [2,3]. These additives react with calcium hydroxide released during cement hydration, forming secondary reaction products that enhance strength and durability and reduce permeability. To further improve the performance, a wide range of additives is used in cementitious materials. Reinforcing additives, including steel, natural, synthetic, or glass fibers, enhance tensile strength, toughness, and crack resistance. Recently, recycled materials such as plastic and glass, as well as agricultural residues, have gained attention for their capacity to strengthen composites while reducing waste [4]. Recycled plastics act as fiber reinforcement, also improving thermal and acoustic insulation, whereas waste glass improves compressive strength and workability [5,6,7]. Pozzolanic materials and plasticizers improve microstructure and long-term durability [8].

The incorporation of biodegradable waste into cementitious materials represents an emerging research area, driven both by the need to reduce environmental impact and the interest in developing sustainable construction composites [1]. Lignocellulosic waste generated by agricultural, forestry, and wood processing industries consists mainly of lignin, cellulose, and hemicellulose. Materials such as rice straw, oil palm residue, hazelnut shells, arhar stalks, coir, bark, cork granules, and bagasse have shown potential as alternative raw materials for cementitious composites [7,9,10,11,12,13,14]. For example, alkali-treated sugarcane bagasse fibers enhance porosity, toughness, and density by improving fiber-matrix adhesion, while NaOH-treated corn straw fibers improve workability. Rice husk and its ash also function as lignocellulosic fillers [15,16]. The morphology and flexibility of these fibers improve mechanical properties such as flexural and tensile strength, ductility, and crack resistance while reducing composite density [17].

Lignocellulosic waste, such as vine shoot waste (VSW), is a potential candidate as a reinforcing fiber for cementitious materials [18]. Additionally, the ash produced from the combustion of VSW can serve as a filler due to its reactive pozzolanic components that participate in secondary reactions with calcium hydroxide released during cement hydration, leading to microstructural densification, reduced porosity, and improved durability [19]. However, the performance of such composites depends on parameters such as combustion temperature, fiber preprocessing, substitution ratio, and compatibility with cement hydration. Although VSW shows significant potential, further research is needed to fully understand the physicochemical interactions between components and prove the benefits in terms of mechanical performance and sustainability [1,20,21].

Lignocellulosic materials influence cement hydration. Polysaccharides can affect the nucleation and development of calcium–silicate–hydrate (C–S–H) and modify the setting time by altering the mechanisms that govern the hydration process. Cellulose provides structural strength and hydrophilicity; hemicellulose acts as a matrix filler; and lignin contributes to rigidity and durability [22,23,24]. Lignin also reduces water demand through hydrogen-bonding interaction and improves dispersion by adsorption onto cement particles and electrostatic repulsion [25,26,27]. Oppositely, the poor dispersibility of cellulose and hemicellulose in aqueous systems can lead to defect formation and mechanical degradation; however, chemical treatments, surface modifications, thermal processing, fiber reinforcement, and nanomaterial incorporation have been shown to enhance interfacial bonding and durability [28,29,30].

Biomass-derived ash, rich in silica and calcium-based phases, is widely used as a mineral additive and can improve durability, reduce cement consumption, and promote the formation of C–S–H gel [31]. Calcium oxide present in ash enhances cementitious behavior by accelerating early-age hydration and improving interfacial bonding. Compared with other biomass-derived ashes such as rice husk, wheat straw, or sunflower residues, vineyard waste-derived ash contains higher CaO content, contributing to accelerated hydration, improved early strength, and superior bonding [32,33].

Vineyard pruning generates large amounts of vine shoot waste (VSW) annually, which is typically treated through burning or composting. The valorization of this biomass and its derived ash in cement composites represents a sustainable waste-management strategy that aligns with circular-economy principles [34,35,36,37]. By incorporating raw VSW, water absorption increases due to its hydrophilic nature, and its performance depends on factors such as density, particle size, dosage, mix design, and processing parameters [38,39]. Although numerous studies have investigated the use of various fillers in cementitious materials [40,41,42], most investigations have focused on specific conditions, such as filler dosage, curing regimes, or material combinations, but a comprehensive evaluation that integrates structural characterization with mechanical performance remains limited. Moreover, despite growing interest in biomass valorization, the use of VSW and its ash in cementitious systems remains largely unexplored [36]. This study addresses this gap by investigating the effects of VSW and its derived ash on the microstructure and mechanical performance of cementitious composites. In this context, the aim of this study was to develop and characterize cement-based composites incorporating VSW and its derived ash, using two particle size fractions, to evaluate their effects on the mechanical and structural properties. The composite materials were characterized using Fourier transform infrared spectroscopy (FT-IR), X-ray diffraction (XRD), scanning electron microscopy (SEM), and nuclear magnetic resonance spectroscopy (NMR), alongside mechanical and durability testing, to assess the feasibility and performance of these composites.

## 2. Results and Discussion

### 2.1. Chemical Composition of VWS and Ash Used for Obtaining the Composite

This study investigated the incorporation of vine shoot waste (VSW) and the ash derived from VSW in cement paste to produce a composite. The chemical composition of VWS and derived ash is presented in Table 1. The parameters include structural components (cellulose, hemicellulose, lignin), proximate analysis (ash, moisture, extractable), elemental composition (C, H, O, S), and inorganic oxides (Na_2_O, MgO, Al_2_O_3_, K_2_O, CaO, and Fe_2_O_3_).

The chemical composition indicates that vine shoot waste (VSW) contains cellulose (36.0 ± 0.23%), hemicellulose (28.0 ± 0.20%), and lignin (28.96 ± 0.23%), which together account for more than 90% of its organic content. This high lignocellulosic content suggests that VSW have good structural integrity, making it suitable as a reinforcing component in cementitious composites. The significant proportion of cellulose contributes to tensile strength, while lignin provides stiffness and resistance to biodegradation, both of which enhance composite performance. The ash content of VSW is relatively low (5.92 ± 0.04%); however, this fraction is significant because it may exhibit pozzolanic properties. This ash incorporated into cement matrices, potentially enhance densification and promote long-term strength development. Moisture content is moderate (8.19 ± 0.01%), indicating that only minimal drying may be necessary before processing to prevent adverse effects on cement hydration. The extractable fraction (3.30 ± 0.04%) is low, suggesting a small amount of soluble organic compounds that could otherwise interfere with setting time or hydration kinetics. Elemental analysis further confirms the organic nature of VSW, with carbon (43.1 ± 0.30%) and oxygen (45.6 ± 0.21%) as the predominant elements. The chemical composition confirms that VSW is a promising raw material for producing both reinforcing fibers and reactive ash, supporting its potential to develop sustainable, enhanced cement-based composites. CaO (11.26%) and MgO (9.67%) are the most abundant oxides. The high content of CaO, MgO, and K_2_O suggest that the ash could be a raw material for clinker production. MgO significantly influences clinker properties such as burnability, phase composition, cement strength, and volume stability [43].

Particle size strongly influences both the physical and chemical properties of composites and is an indicator of composite quality and performance. It governs the flowability and compaction. Small particles with high surface area typically improve emulsion and suspension stability. The fine VSW particles had an average size of 23.4 µm, whereas coarse VSW particles measured 330 µm; both showing irregular, fibrous morphologies. In contrast, the ash particles obtained after combustion of VSW were more compact and angular in appearance, with mean particle sizes of 38.1 µm for the fine ash fraction and 128 µm for the coarse fraction.

### 2.2. Chemical and Structural Characterization of the Composite

The investigations were conducted over 28 days, a period corresponding to near-complete cement hydration and sample stability. The incorporation of VSW and ash positively affects the microstructure of the cement paste. However, this enhancement requires a higher water content compared to conventional cement paste.

#### 2.2.1. Fourier Transform Infrared Spectroscopy (FT–IR)

**Figure 1:**
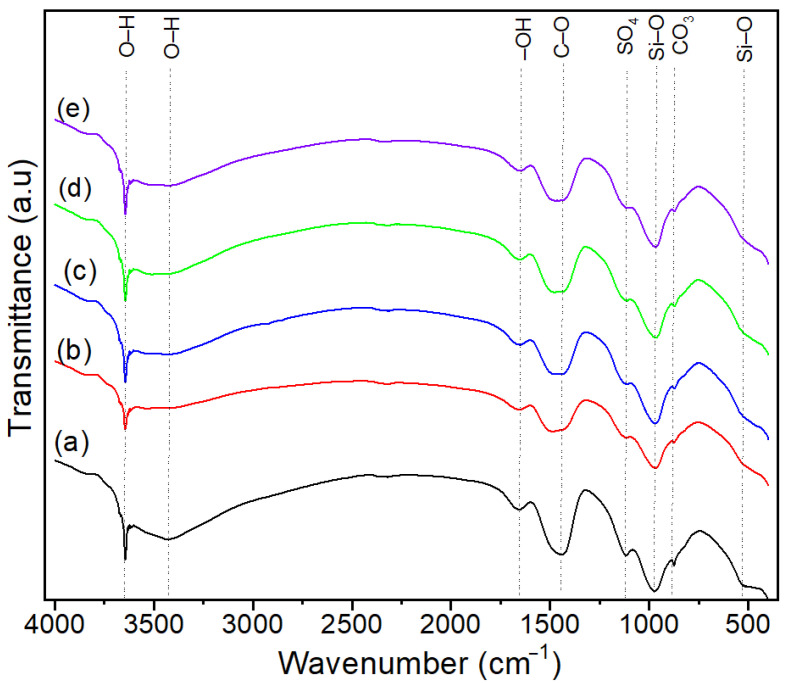
FT–IR spectra of (a) (CP), (b) CP–FW, (c) CP–FA, (d) CP–CW, and (e) CP–CA samples.

The FT-IR spectra of the cement paste and the composite samples incorporating VSW and VSW-derived ash are presented in Figure 1. All spectra show the characteristic bands for hydrated Portland cement. The broad band observed at 3400–3500 cm^−1^ and the smaller peak at ∼1640 cm^−1^ correspond to the stretching and bending vibrations of –OH and H–O–H bonds in adsorbed and structural water [44,45]. The intensity of these bands decreases in composite samples, indicating a reduction in absorbed water content. The band at ∼3640 cm^−1^ indicates the presence of Ca(OH)_2_, but its relatively low intensity suggests that free lime has been converted into carbonates [44,46]. The carbonation of Ca(OH)_2_ during curing and exposure to atmospheric CO_2_ is indicated by the intense absorption band at ∼1420 cm^−1^ specific to carbonates [45]. The presence of ettringite traces is indicated by the small peak at ∼1100 cm^−1^ specific to SO_4_^2−^ vibration [47]. Intense bands at ∼960 cm^−1^ and 530 cm^−1^ are associated with Si–O stretching and bending and confirm the formation of calcium–silicate–hydrate, the primary binding phase in cementitious materials. However, the strength of cement-based materials is also influenced by factors such as the formation of ettringite, the persistence of unreacted clinker, the porosity and connectivity of the pore network [45,48,49,50]. The incorporation of lignocellulosic waste does not appear to hinder the synthesis of C–S–H, as confirmed by the same intensity of the band at ∼960 cm^−1^ in both the composites and the cement paste.

#### 2.2.2. X–Ray Diffraction (XRD)

**Figure 2:**
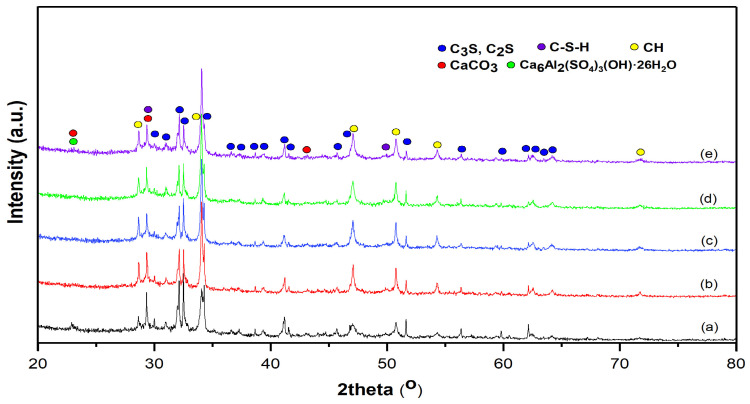
XRD patterns of (a) CP, (b) CP–FW, (c) CP–FA, (d) CP–CW, and (e) CP–CA samples.

Figure 2 shows the XRD of the cement paste (CP) and the composite samples incorporating VSW and its derived ash. The presence of calcite is explained by the carbonation reaction between calcium hydroxide (C–H) and atmospheric carbon dioxide (CO_2_). Additionally, traces of ettringite are observed in all samples, indicating phase reversal. The presence of belite (C_2_S) and alite (C_3_S) further suggests that the clinker phase transformation is incomplete. Overall, the same crystalline phases are observed in both the CP and composites [51]. The diffraction peaks associated with unreacted clinker minerals are still present in the cement paste containing vine shoot waste, indicating that not all of the clinker has transformed into hydration products such as calcium silicate hydrate (C–S–H) [52]. Moreover, experimental factors such as limited curing time, restricted moisture availability, or carbonation during sample preparation may have contributed to the retention of crystalline clinker peaks. These observations suggest that the VSW may retard or interfere with the complete hydration of the cement clinker, the main reactive component of the cement [53]. Under normal curing conditions, lignocellulosic waste does not directly react with primary cement phases (C_3_S and C_2_S). However, interactions can occur through physical hindrance or adsorption of ions at the fiber-matrix interface, which can slow early hydration. After calcination at 550 °C, any amorphous organics that are undetectable by XRD are fully oxidized. Therefore, the absence of organic compounds confirms that the vine shoot waste contains no organic matter.

The composition of the crystalline phases of the investigated samples, as determined using the RIR method, is summarized in Table 2.

The higher C–H content in the composites suggests that the additives, in both their raw and calcined forms, may retard the subsequent consumption of C–H during early hydration, either by hindering ion diffusion (VSW) or by delaying pozzolanic reaction (ash). In contrast, the amount of C–S–H remains nearly unchanged across all samples, indicating that the primary C–S–H-forming hydration reactions proceed at comparable extents within the studied curing period. The resulting ash can act as a pozzolanic additive that reacts with C–H to densify the microstructure, thereby enhancing the mechanical properties and durability of the material [51]. The degree of crystallinity (DC) decreases from 73.8% in CP to about 70% in all modified pastes. This slight reduction is consistent with the partial substitution of clinker minerals by less crystalline components (VSW) or by ash with a higher amorphous content, indicating that the additives introduce a slight increase in amorphous phases without altering the crystalline hydration products.

#### 2.2.3. Scanning Electron Microscopy Energy Dispersive X–Ray Spectroscopy (SEM–EDX)

**Figure 3:**
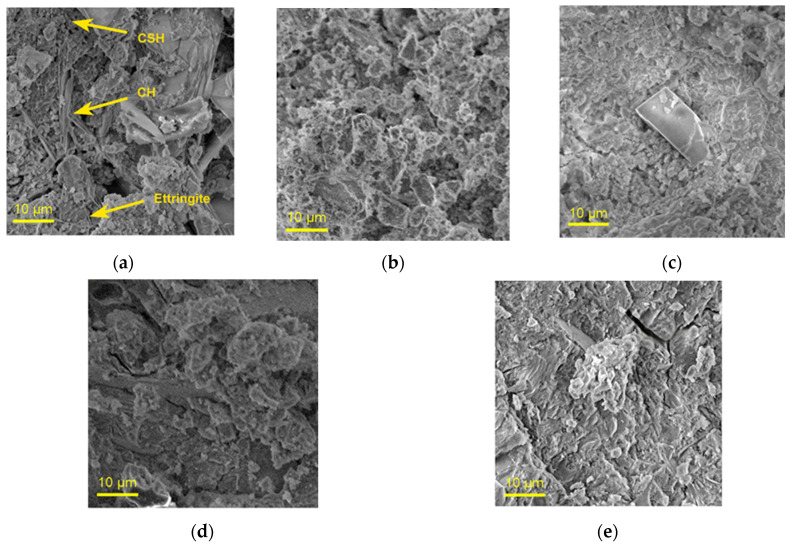
SEM images of (**a**) CP, (**b**) CP–FW, (**c**) CP–FA, (**d**) CP–CW, and (**e**) CP–CA samples.

**Figure 4:**
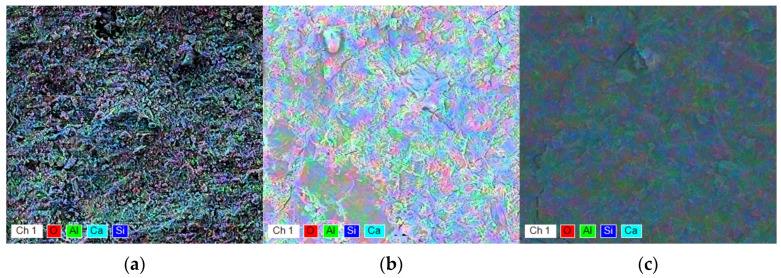
EDX elemental maps of (**a**) CP, (**b**) CP–CW, and (**c**) CP–CA samples.

SEM-EDX was used to characterize the surface topography and elemental composition of the samples. The surfaces of the samples at high magnification are illustrated in Figure 3, whereas the EDX elemental maps are shown in Figure 4. All samples present surface irregularities characterized by the presence of voids and cracks, suggesting structural disruption. Figure 3a, which shows the surface of CP, was used to highlight the characteristic phases of cement paste. The replacement of a small percentage of CP with fine VSW leads to an increase in C–S–H, a decrease in C–H units, and the coverage of all sample surfaces with tiny needles of ettringite. This behavior must be due to the organic phase of the wood, which alters the dynamic interactions of water with the sample. The use of fine ash leads to a decrease in C–H and ettringite phases and an increase in C–S–H gel. From a chemical perspective, a similar behavior is noted with the use of coarse VSW and ash. Additionally, higher structural irregularities become apparent as the particle size of the VSW and ash increases.

The presence of voids and cracks typically indicates reduced mechanical strength of the samples. The obtained images suggest that the incorporation of coarse wood and ash may compromise mechanical properties. However, fine ash appears to retain mechanical strength comparable to that of pure CP, indicating its potential as a suitable additive.

To gain further insights into the composition of the samples and the distribution of elements on their surfaces, EDX analysis was conducted. The EDX maps of the samples are illustrated in Figure 4, with detailed results provided in Table 3. The samples show similar overall chemical compositions, suggesting that the morphological differences observed in Figure 3 are likely due to variations in the relative proportions of the structural units present.

The EDX maps, colored according to elemental composition, confirm the findings of the XRD analysis. Notably, the green color representing aluminum (Al) is more prevalent in the CP–CW and CP–CA samples compared to the CP sample, indicating a potential increase in ettringite content in these samples, consistent with the XRD results. Additionally, the more intense light blue areas in the CP–CW and CP–CA samples suggest a higher concentration of calcium hydroxide (C–H) relative to the CP sample.

In the CP–CW sample, oxygen appears to be more abundant, possibly because the organic components of wood lose water more slowly than those of the other samples, resulting in a slower hydration process. Overall, the EDX maps corroborate results obtained from other methods and provide a clearer understanding of the phase distribution across the surface of the samples.

#### 2.2.4. Nuclear Magnetic Resonance (NMR) Spectroscopy

**Figure 5:**
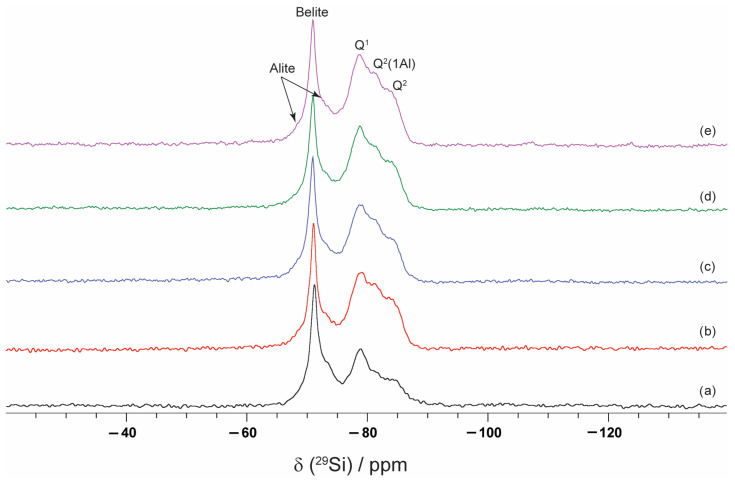
^29^Si NMR spectra of (a) CP, (b) CP–FW, (c) CP–FA, (d) CP–CW, and (e) CP–CA samples.

**Figure 6:**
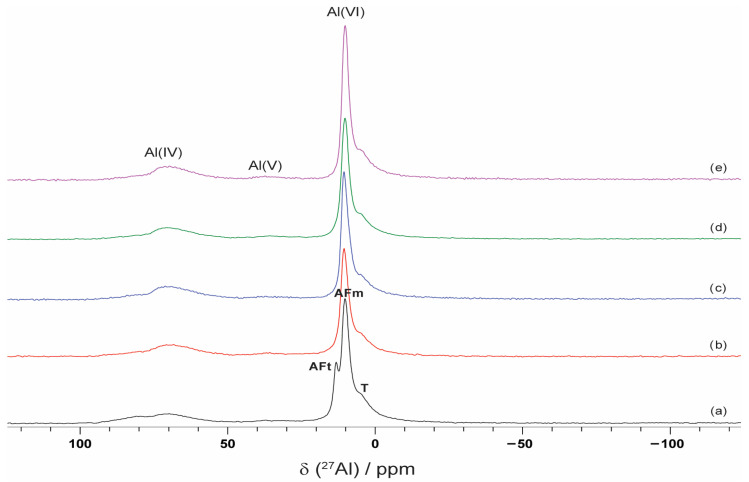
^27^Al NMR spectra of (a) CP, (b) CP–FW, (c) CP–FA, (d) CP–CW, and (e) CP–CA samples.

The ^29^Si and ^27^Al NMR spectra are presented in Figure 5 and Figure 6. The ^29^Si spectra for all samples exhibit a spectral line at −71.3 ppm corresponding to belite superimposed on two overlapping resonances from alite primary phase [54]. A second spectral line, ranging from −75 to −90 ppm, corresponds to C–S–H. This spectral line includes resonances from Q^1^ (−78.8 ppm), Q^2^ (1Al) (−81.4 ppm), and Q^2^ (−84.3 ppm) structural elements. The presence of VSW enhances cement hydration, leading to the formation of larger quantities of hydration products and an increase in the contents of Q^2^(1Al) and Q^2^ species (compared with Q^1^).

The ^27^Al spectra show two distinct regions, corresponding to Al(IV) and Al(VI), and a very small peak for Al(V) [55]. The two peaks present in region Al(IV) are one at 70.8 ppm, corresponding to the bridging sites in C–S–H, and another at 80.5 ppm, associated with unhydrated clinker. For the CP sample, three spectral lines are presented in the Al(VI) region: one at 13.2 ppm, corresponding to the AFt phase; another at 10.2 ppm, related to the AFm phase present in all spectra; and a third at 5 ppm, linked to the third aluminate hydrate (T), also observed in all spectra. No detectable signals for ^27^Al and ^29^Si appear in the nuclear magnetic resonance spectra of the ash sample.

#### 2.2.5. TGA of Composites

**Figure 7:**
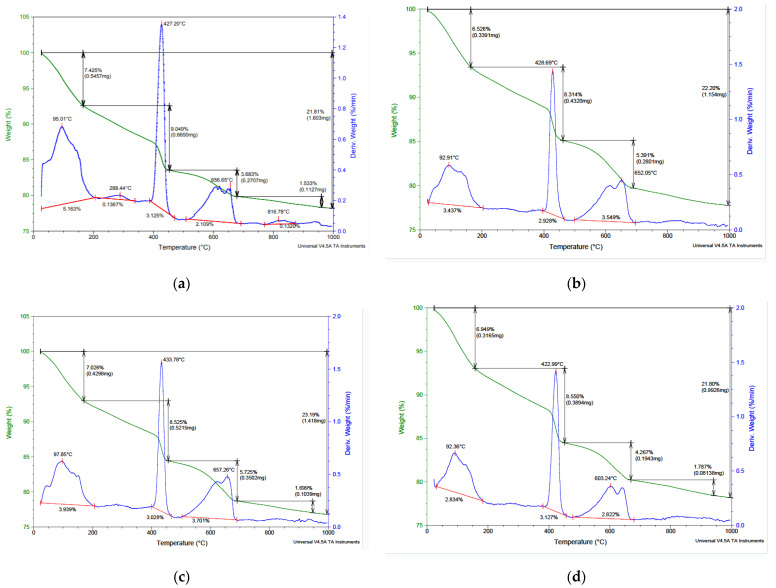
The TGA/DTG curves of the obtained composites: (**a**) CP–FW, (**b**) CP–FA, (**c**) CP–CW, and (**d**) CP–CA.

The TGA/DTG curves of the cement composites are presented in Figure 7. The TGA curves of all samples exhibited three peaks at approximately 95, 430, and 650 °C. The TGA curves of the composites reveal five distinct effects: (i) 100−150 °C correspond to the evaporation of water; (ii) 150–250/250–350 °C are associated with the decomposition of organic components from biomass, particularly hemicellulose, which degrades around 200–315 °C; (iii) the 300–400 °C range corresponds to the degradation of cellulose and lignin, as well as the dehydration of some C–S–H hydrates; (iv) the 400–520 °C range is attributed to the dehydroxylation of portlandite; and (v) the 600–800 °C range corresponds to the decarbonation of CaCO_3_.

TGA of CP–FW (Figure 7a) shows significant mass loss between 200 and 450 °C, corresponding to the thermal decomposition of lignocellulosic components. Due to the fine particle size, thermal decomposition overlaps with C–S–H dehydration. The peak corresponding to portlandite indicates the presence of Ca-based hydration products. The presence of lignocellulosic biomass increases the formation of Ca hydrates, as observed between 600 and 800 °C. The TGA of CP–CW (Figure 7c) shows similar decomposition patterns but is less uniform. The peak observed around 288 °C corresponds to C–S–H hydrates and the decomposition of remaining organic compounds. The peak at 427 °C corresponds to the decomposition of portlandite, indicating a significant amount of Ca(OH)_2_ in the composite [56]. Additionally, the peak at 656 °C indicates the decomposition of C–S–H and carbonate complexes. A clear decarbonation peak at 817 °C corresponds to the carbonation of portlandite. The TGA curve reflects cement hydration behavior, with well-defined dehydration, portlandite decomposition, and calcite decarbonation.

The TGA/DTGA curves of the fine and coarse ash composites (Figure 7b,d) show the decomposition of biomass and portlandite. The TGA of fine VSW confirms its non-pozzolanic character. Additionally, the significant carbonate decomposition indicates enhanced carbonation induced by the porous structure of VSW. VSW acts as an organic filler, modifying the microstructure and promoting carbonation, contributing to hydration [57].

TGA curve of CP–FA shows minimal organic decomposition, prominent portlandite decomposition, and decarbonation. The mass losses confirm a high carbonate content whereas the curve suggests limited pozzolanic consumption. The TGA of CP–CA shows a higher mass loss during decarbonation, indicating a higher carbonate content. Overall, fine ash is more favorable due to its filler effect, whereas coarse ash acts as an inert additive and promotes carbonation [58].

### 2.3. Mechanical Properties

**Figure 8:**
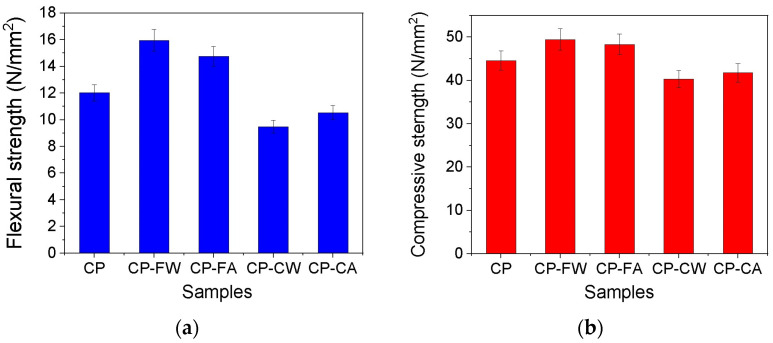
Mechanical properties: (**a**) flexural strength and (**b**) compressive strength.

The flexural and compressive strength of the CP, CP–CW, CP–CA, CP–FW, and CP–FA samples are presented in Figure 8. The mechanical properties decrease in the presence of coarse VSW (CP–CW) and coarse ash from VSW (CP–CA), while they increase in the case of fine VSW (CP–FW) and fine ash from VSW (CP–FA) compared with CP. Similar results were obtained by Tayeh et al. and Dos Santos et al., who used rice husk waste and short sugarcane bagasse fibers, respectively [9,10].

The improved structure of the cement pastes with 1% VSW or 1% ash from VSW is supported by flexural and compressive strength results. In the presence of fine particles of VSW and ash from VSW, the mechanical properties increase compared to the coarse particles of VSW and ash from VSW. The water/cement ratio used in this case is 0.4. The highest flexural strength (15.96 N/mm^2^) is obtained for CP–FW, followed by CP–FA (14.76 N/mm^2^), and the compressive strength is highest (49.45 N/mm^2^) in CP–FW, followed by CP–FA (48.32 N/mm^2^).

CP–FW and CP–FA are samples that incorporate microfibers of fibrous particles, whereas CP–CW and CP–CA contain coarse particles of larger dimensions. The observed enhancement in mechanical properties, specifically flexural and compressive strength, in the samples with fine fractions indicates that these fine particles function as reinforcing agents, analogous to conventional fibers in composite materials.

## 3. Materials and Methods

### 3.1. Chemicals and Reagents

All chemicals used in the experiments were of analytical reagent grade and were purchased from Merck (Darmstadt, Germany). Commercially available white cement CEM I 52.5 R containing 95–100% clinker and 0–5% auxiliary components, also known as Portland cement, was used for all tests. The VSW was procured from the “Ion Ionescu de la Brad” research station of the University of Agricultural Sciences, Iasi, Romania.

### 3.2. VSW Treatment

**Figure 9:**
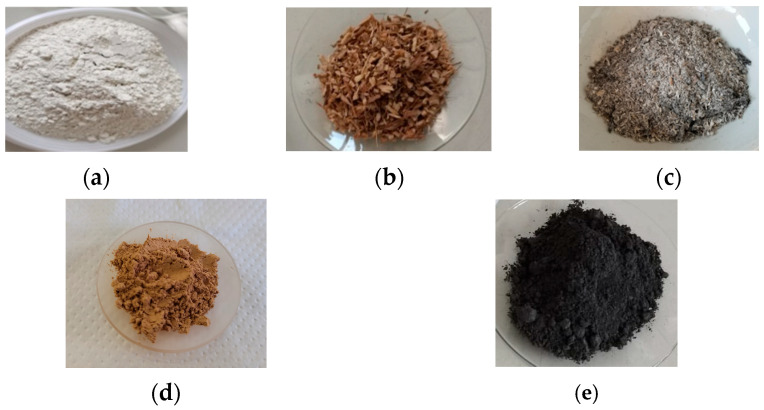
Materials: (**a**) white cement, (**b**) coarse VSW, (**c**) coarse ash derived from VSW, (**d**) fine VSW, and (**e**) fine ash derived from VSW.

The VSW collected after the pruning operations was oven-dried at 60 °C for 24 h. The ash derived from VSW was obtained after the thermal treatment of VSW at 550 °C for 5 h. The dried VSW and ash from VSW were ground and sieved to obtain two particle sizes: fine and coarse. Figure 9 presents the materials used for the preparation of the samples.

### 3.3. Cement Composite and Paste Preparation

The cement grains were first dry-mixed with VSW or ash at a water-to-cement ratio of 0.4 and a VSW or ash dosage of 1% (Table 4) till a homogenous mixture was obtained. The low dosage (1%) of VSW and ash incorporated into cement was selected based on literature studies indicating that additions of low amounts of biomass and biomass-derived ash enhance cement properties while minimizing risks to hydration and microstructure [1]. Then, distilled water was added, and the mixing was continued using an electrical mixer with a rotational frequency of 700 rpm for 5 min. Distilled water was used instead of tap water to ensure controlled and reproducible results during composite preparation. Tap water typically contains dissolved ions such as Ca^2+^, Mg^2+^, Cl^−^, SO_4_^2−^ that can chemically interact with cement hydration products and potentially alter the strength and durability of the composite, whereas distilled water is free of these impurities, ensuring that hydration reactions proceed only between the intended mix components and removes uncontrolled variables that could affect cement hydration and the final mechanical properties [59]. The resulting homogenous paste was then poured into standard molds. The filled molds were stored in a climate-controlled closed chamber in which temperature (20 °C) and humidity (45%) were maintained constant. After 24 h, the samples were demolded and transferred to a water bath until testing. White cement and cement paste (CP) were used as controls when comparing the properties of cement composites.

### 3.4. Chemical Characterization of Raw Materials

The holocellulose content (a mixture of cellulose and hemicellulose) was obtained after refluxing the VSW with NaClO_2_ in 10% acetic acid at 75 °C for 1 h. The resulting holocellulose was filtered, dried at 105 °C for 24 h, and weighed. Cellulose content was measured as the fraction of holocellulose insoluble in 17.5% NaOH. Specifically, holocellulose was treated with 25 mL of 17.5% NaOH at 20 °C for 40 min, then washed, dried at 105 °C for 48 h, and weighed. Hemicellulose content was calculated as the difference between holocellulose and cellulose. Lignin content was determined as the residue insoluble in 72% H_2_SO_4_. For this, the dried biomass was treated with 72% H_2_SO_4_ at 20 °C for 4 h, diluted with 500 mL distilled water, and refluxed for another 4 h. The residue was filtered, washed, dried at 105 °C for 24 h, and weighed. Lignin percentage was calculated as the ratio of lignin mass to the initial sample mass [12]. Ash and moisture were analyzed gravimetrically. The moisture content was determined by drying for 24 h at 105 °C in an oven (UFE 400 Memmert, Memmert, Schwabach, Germany) to a constant weight. The ash content was determined after the incineration of samples at 550 °C in a LT9/1300/C450 Muffle Furnace (Nabertherm, Lilienthan, Germany). Elemental analysis content was determined using a Flash EA 2000 CHNS/O analyzer (Thermo Fisher Scientific, Waltham, MA, USA). The chemical characterization of major elements and their corresponding oxides (Na_2_O, K_2_O, CaO, Al_2_O_3_, Fe_2_O_3_, and MgO) was carried out. The major elements were analyzed using an Optima 5300 DV inductively coupled plasma optical emission spectrometer (ICP-OES; Perkin Elmer, Norwalk, CT, USA).

### 3.5. Structural Characterization of Composites Preparation

#### 3.5.1. FT-IR Analysis

Before FT-IR analysis, samples were dried at 105 °C to remove residual moisture and ground into fine, homogeneous powders. Pellets were prepared by pressing a 1:100 (*w*/*w*) sample-to-KBr homogenous mixture using a 13 mm die and a 15-ton hydraulic press (CrushIR, PIKE Technologies, Madison, WI, USA). FT-IR spectra were collected in transmission mode over the 4000–400 cm^−1^ range with a spectral resolution of 2 cm^−1^ using a Spectrum BX II spectrometer (PerkinElmer, Waltham, MA, USA).

#### 3.5.2. XRD Analysis

The XRD patterns were obtained using a D8 Advance diffractometer (Bruker, Karlsruhe, Germany) with CuKα1 radiation (λ = 1.5418 Å), operated at 40 kV and 40 mA. Semi-quantitative analysis was conducted using the reference intensity ratio (RIR) method [60], while the degree of crystallinity was determined as the ratio of the total area of the diffraction peaks to the combined area of the diffraction peaks and amorphous halos.

#### 3.5.3. SEM Analysis

The SEM images were recorded using a VEGAS 3 SBU (Tescan, Brno–Kohoutovice, Czech Republic) scanning electron microscope equipped with a Quantax EDX XFlash (Bruker, Karlsruhe, Germany) detector. The particle size of the VSW and ash was determined after sieving by using SEM equipment (Tescan, Brno–Kohoutovice, Czech Republic).

#### 3.5.4. Solid-State ^27^Al and ^29^Si Nuclear Magnetic Resonance (NMR) Spectroscopy

The MAS NMR spectra were acquired using a 500 MHz Bruker Avance III solid-state wide-bore spectrometer (Bruker BioSpin GmbH, Aachen, Germany), functioning at Larmor frequencies of 99.36 MHz for ^29^Si and 130.32 MHz for ^27^Al. The ^29^Si MAS NMR spectra were acquired with a 4 mm Bruker MAS probe head, with materials encapsulated in ZrO_2_ rotors, which were rotated at 7 kHz. The method employed a collection of 15,000 free induction decays (FIDs) with a 5 s recycle delay within a one-pulse proton High Power Decoupling process. The ^29^Si spectra were calibrated to tetramethylsilane (TMS) through an indirect approach, employing sodium–3–(trimethylsilyl)–propane–1–sulfonate (DSS) at 1.46 ppm as an external reference. A 2.5 mm probe head Bruker MAS and ZrO_2_ rotors spun at a frequency of 25 kHz were utilized for the signal recording of ^27^Al MAS NMR spectra. It employed a 3000 (FIDs) collection with a 1.5 ms pulse width (one-pulse sequence) and a recycle delay of 1 s. The aluminum in a 1 M aqueous solution of Al(NO_3_)_3_, as an external standard, was used for the signal calibration at 0 ppm for ^27^Al spectra.

#### 3.5.5. Thermal Analysis

The thermal decomposition and derivative thermogravimetric (DTG) behavior of the samples was analyzed using a TA Instruments SDT Q600 (TA Instruments, New Castle, DE, USA). Measurements were conducted from 30 to 1000 °C at a heating rate of 10 °C min^−1^. Dried samples weighing 8.0 ± 0.3 mg were tested under a nitrogen atmosphere. All experiments were repeatable, with peak temperature values showing only minimal standard deviation.

### 3.6. Mechanical Analysis

The mechanical parameters were assessed using 40 × 40 × 160 mm prisms for flexural strength and 40 × 40 × 40 mm cubes for compressive strength [61] with a UTCM–3742 15/250 kN automatic testing machine (Utest Material Testing Equipment, Ankara, Turkey). Flexural strength was measured in a one-point loading setup by applying a load in the perpendicular direction at the midpoint of the specimen placed on two supported beams. Compressive strength was determined by positioning the cubic specimen on a fixed platen measuring 40 × 40 × 40 mm while applying a load from above using a platen of the same size.

## 4. Conclusions

This study evaluated the effects of incorporating VSW and its ash derivative, at both coarse and fine particle sizes, on the hydration, microstructure, and mechanical properties of cement paste at 28 days. The spectroscopic and diffraction analyses (FT-IR, XRD, NMR) showed that the chemical hydration pathways of the studied Portland cement remain unaltered by the addition of VSW. The SEM-EDX analysis revealed that fine VSW particles (<100 μm) provide a more uniform microstructure, which is associated with enhanced mechanical strength. The incorporation of fine VSW constitutes a viable route for valorizing agricultural by-products in cement-based materials, leading to an improvement in the composite’s mechanical strength and microstructural density. Conversely, coarse particles (1–4 mm) produce a less homogeneous microstructure with inherent weaknesses, leading to a reduction in both flexural and compressive strength. The present study demonstrates the feasibility of incorporating VSW into cementitious materials, the applied characterization techniques confirming measurable changes in the material’s properties. These findings provide preliminary evidence that agricultural by-products can be used in construction materials. However, the broader implications for environmental sustainability and for the integration of the circular economy require further validation. Therefore, future research should expand the dosage range, including long-term durability testing, and fully evaluate the potential of VSW as a sustainable additive in cement composites.

## Figures and Tables

**Table 1:** Chemical composition of VSW and VSW-derived ash used as raw materials for the composite preparation (nd = not determined) (samples expressed as averages ± standard deviation (*n* = 3).

Parameters	Raw Materials	
	VSW (%)	Ash (%)
Cellulose	36.0 ± 0.23	nd
Hemicellulose	28.0 ± 0.20	nd
Lignin	28.9 ± 0.23	nd
Ash	5.92 ± 0.04	nd
Moisture	8.19 ± 0.01	nd
Extractable	0.62 ± 0.04	nd
C	43.1 ± 1.30	60.7 ± 2.1
H	6.23 ± 0.01	7.31 ± 0.01
N	1.60 ± 0.05	1.73 ± 0.06
O	45.6 ± 0.21	30.2 ± 0.3
S	<0.01	<0.01
Na_2_O	nd	0.08 ± 0.001
MgO	nd	9.67 ± 0.74
Al_2_O_3_	nd	0.04 ± 0.002
K_2_O	nd	6.99 ± 0.51
CaO	nd	11.3 ± 1.1
Fe_2_O_3_	nd	0.09 ± 0.004

**Table 2:** Phase fraction (%) and the degree of crystallinity (DC, %) of CP, CP–FW, CP–FA, CP–CW, and CP–CA samples.

Samples	CP	CP–FW	CP–FA	CP–CW	CP–CA
C_2_S	21.5 ± 3.2	16.1 ± 2.4	16.3 ± 2.4	16.0 ± 2.4	16.1 ± 2.4
C_3_S	38.9 ± 5.8	34.1 ± 5.1	34.3 ± 5.1	34.0 ± 5.1	34.2 ± 5.1
C–H	11.6 ± 1.7	24.0 ± 3.6	24.2 ± 3.6	23.9 ± 3.6	24.1 ± 3.6
C–S–H	6.4 ± 1.0	5.9 ± 0.9	5.9 ± 0.9	5.8 ± 0.9	5.8 ± 0.9
Calcite	7.1 ± 1.1	7.8 ± 1.2	7.8 ± 1.2	7.6 ± 1.1	7.7 ± 1.2
Ettringite	14.5 ± 2.2	12.1 ± 1.8	11.5 ± 1.7	12.7 ± 1.9	12.1 ± 1.8
DC	73.8	70.8	71.2	70.1	70.5

**Table 3:** Elemental composition of CP, CP–FW, CP–FA, CP–CW, and CP–CA samples.

Sample	O (%)	Ca (%)	Si (%)	Al (%)	S (%)
CP	47.94	40.45	9.83	1.78	-
CP–FW	46.77	41.31	8.84	1.74	1.33
CP–FA	46.27	41.46	9.49	1.59	1.19
CP–CW	50.65	38.58	9.06	1.71	-
CP–CA	53.20	35.22	9.91	1.68	-

**Table 4:** Composites preparation.

Composite	Raw Materials	Water–to–Cement Ratio	Vine Shoot Waste–to–Cement Ratio
CP	CP	0.40	–
CP–FW	CP + fine VSW	0.40	0.01
CP–FA	CP + fine ash of VSW	0.40	0.01
CP–CW	CP + coarse VSW	0.40	0.01
CP–CA	CP + coarse ash of VSW	0.40	0.01

## Data Availability

The original contributions presented in this study are included in the article. Further inquiries can be directed to the corresponding authors.

## Abbreviations

The following abbreviations are used in this manuscript:

CP–FW	Cement paste with fine vine shoot waste
CP–FA	Cement paste with fine ash derived from vine shoot waste
CP–CW	Cement paste with coarse vine shoot waste
CP–CA	Cement paste with coarse ash derived from vine shoot waste
EDX	Energy dispersive X–ray spectroscopy
FT-IR	Fourier transform infrared spectroscopy
NMR	Nuclear magnetic resonance spectroscopy
SEM	Scanning electron microscopy
XRD	X-ray diffraction
VSW	Vine shoot waste
CH	Calcium hydroxide
CP	Cement paste
C_2_S	Belite
C_3_S	Alite
